# Analysis of the Improvement of Engineering Mechanics Experimental Methods Based on IoT and Machine Learning

**DOI:** 10.3390/s23073416

**Published:** 2023-03-24

**Authors:** Yi Sun, Dongfa Sheng, Dewen Liu

**Affiliations:** Institute of Civil Engineering, Southwest Forestry University, Kunming 650224, China

**Keywords:** intelligent sensor, Internet of Things application, machine learning algorithm, LE-LPCCA model, engineering test optimization

## Abstract

With the rapid development of sensor technology, machine learning, and the Internet of Things, wireless sensor networks have gradually become a research hotspot. In order to improve the data fusion performance of wireless sensor networks and ensure network security in the event of external attacks, this paper proposes a wireless sensor optimization algorithm model, involving wireless sensor networks, the Internet of Things, and other related fields. This paper first analyzes the role of the Internet of Things in wireless sensor networks, studies the localization mechanism and hierarchy of the Internet of Things based on wireless sensor networks, and improves the LE-RLPCCA (Position Estimation Robust Local Retention Criteria Correlation Analysis) localization algorithm model based on sensor grids. This paper discusses the problems of machine learning in wireless sensor networks, constructs a sensor-based machine learning model, and designs a data fusion algorithm for a wireless sensor networks’ machine learning model. The application of wireless sensors in engineering mechanics experiments is summarized, and the optimization algorithm model of the wireless sensor in engineering mechanics experiments is proposed. The analysis results show that the average accuracy of the DKFCM-FSVM (Density aware Kernel-based Fuzzy C-means Clustering algorithm Fuzzy Support Vector Machine) algorithm in detecting five behaviors is 0.997, 0.992, 0.904, 0.996, and 0.946, respectively, and the accuracy in detecting different behaviors is the best, 0.005, 0.01, 0.003, and 0.006 respectively. It achieves the lowest false positive rate in the detection of different behaviors, and the average false positive rate is 0.004, 0.003, 0.003, 0.008, and 0.005, respectively, which shows that the DKFCM-FSVM algorithm model of wireless sensor networks in engineering mechanics experiments is the optimal solution. The work of this paper has good reference value for the application of wireless sensor networks and the optimization of engineering mechanics experimental methods and is helpful for further research of sensor technology.

## 1. Introduction

With the rapid development of sensor technology, machine learning, and the Internet of Things, wireless sensor networks have gradually become a research hotspot. Because of its broad application prospects, many scientists have conducted in-depth research on wireless sensor technology. A number of challenging issues have been raised for logical and technological specialists at the fundamental hypothesis and design innovation levels. Remote sensor systems (WSNs) are a multi-jump self-organized framework formed by a large number of sensor hubs located within the observing range communicating with each other, and they plan an important role the Internet of Things [1]. With the development of remote communication, sensor innovation, inserted application, and microelectronics innovation, remote sensor systems can obtain the data individuals require at any time, any place, and under any natural conditions, laying the basis for the improvement of the Internet of Things (loT). Due to the specialized preferences of self-organization, fast arrangement, high fault tolerance, and solid concealment, remote sensor systems are exceptionally appropriate for war zone target areas [2], physiological information collection [3], the brilliantly transportation framework [4], sea investigation [5], and numerous other sites.

In recent years, sensors, radio frequency identification tags (RFID), machine-to-machine (M2M), and other related technologies have developed rapidly. The Internet of Things (IoT) has become a hot technology in the academic research field and industrial manufacturing field and will play an important role in the new Internet in the future [6]. As an important part of the Internet of Things for data discernment, remote sensor systems play a progressively critical role. A wireless sensor arrangement can be a disseminated remote organization, which is composed of a large number of low-power sensor hubs deployed within the detecting range through remote communication joins. A sensor hub is a kind of miniaturized scale computing unit, which has the characteristics of low capacity and constrained computing control and battery control. Due to the openness of remote systems and the impediments of sensor hubs, remote sensor systems are confronting an assortment of security threats [7]. The presentation of key administration and personal confirmation components as the primary line of defense to secure remote sensor systems can viably guard against assaults from outside the remote sensor networks [8]. Due to the restrictions of remote sensor systems, conventional intrusion detection innovations cannot be straightforwardly connected to remote sensor networks [9]. The research into an intrusion detection technology suitable for wireless sensor networks has become a research hotspot, which has attracted the extensive attention of experts and scholars worldwide.

While the existing research on the detection algorithms of engineering mechanics tests has achieved some results, there are also some problems: the detection rate for known attack types is high, but the effect of the unknown attacks is poor. Although wireless sensor network algorithms or models can be applied to non-ideal data with certain errors, their effects are not satisfactory when erroneous data exist. Although they can be detected in advance and eliminated by some means, WSN has strict restrictions and requirements on the energy and storage of sensor nodes, and simple detection algorithms cannot guarantee that these erroneous data can be eliminated completely.

To improve the detection rate, some detection algorithms introduce complex mathematical models, but these algorithmic models are not applicable to the application scenario of external behavioral intrusion into sensor networks. For the problem of the low performance of wireless sensor data fusion, the localization mechanism of the IoT based on wireless sensor networks is analyzed herein. An LE-LPCCA localization algorithm based on wireless sensor networks is designed. Then, an improved LE-RLPCCA localization algorithm model is proposed. A machine learning model for wireless sensor networks is constructed on this basis, and finally, the validity of the model is verified using engineering mechanics tests. The results show that the behavior detection accuracy of the DKFCM-FSVM algorithm is higher, while the false positive rate and negative rates are both lower, indicating that the algorithm is more suitable for unbalanced datasets and can effectively deal with outliers and noisy data. In engineering mechanics experiments, the DKFCM-FSVM can be applied to real WSNs scenarios, thus improving the ability of WSNs to resist attacks inside the network. In addition, wireless sensor network algorithms have achieved remarkable success in pattern recognition and image processing, providing the possibility of the optimization of intrusion detection.

## 2. Related Work

Experts and scholars have conducted a lot of research on wireless sensor algorithms in engineering mechanics. Zhang et al. [10] introduced the rough set method to improve the sensor detection algorithm model based on artificial immunity, combined anomaly detection and misuse detection, and proposed an intrusion detection method, which achieved vaccine injection without terminating the intrusion detection behavior. To solve the problem of the poor intrusion detection performance in resource-constrained wireless sensor networks, Luo et al. [11] proposed an intrusion detection algorithm based on machine learning for wireless sensor networks. This intrusion detection algorithm had the characteristics of a high detection rate and low computational complexity and can be applied to the WSN application scenario. Jiang et al. [12] combined the adaptive AP algorithm with a clustering algorithm, proposed an adaptive AP clustering algorithm, and applied it to intrusion detection. This algorithm reduced the number of samples for clustering and reduced the clustering time. Liu [13] proposed an improved algorithm to overcome the shortcomings of traditional fuzzy sensor clustering algorithms, using the Mercer kernel to define the objective function of the FCM algorithm to improve the optimization ability of FCM. Zhang et al. [14] proposed a hybrid multilevel sensor detection algorithm model to solve the problem of the low detection rate of Probe (probing) and U2R (user robot) sensors. Makarfi et al. studied the Internet of Things (IoT) network using reconfigurable intelligent surfaces (RIS) over generalized fading channels [15]. Guan et al. proposed a location-free shortest path reliable and energy-efficient stress-based routing (SPRE-PBR) protocol and designed the SPRE-PBR algorithm to control the path selection and reduce unnecessary forwarding based on the routing cost calculation and the optimal shortest path algorithm [16]. Mao et al. proposed a data knowledge representation method of fuzzy expert knowledge combining the structural learning ability of preprocessing collaborative fuzzy clustering and the Takagi–Sugeno–Kang model [17]. Jiao et al. proposed an energy balance routing protocol with depth control. This protocol was able to adjust the depth of low energy nodes and exchange low energy nodes with high energy nodes to ensure consistent energy utilization [18]. Subramani et al. designed a metaheuristic UWSN routing protocol called MCR-UWSN. The goal of the MCR-UWSN technology was to select a set of effective cluster heads and route to the destination [19]. The comparison of the above research methods is shown in Table 1.

## 3. Wireless Sensor Network Model for the Internet of Things

### 3.1. The Role of the Internet of Things in Wireless Sensor Networks

**Figure 1:**
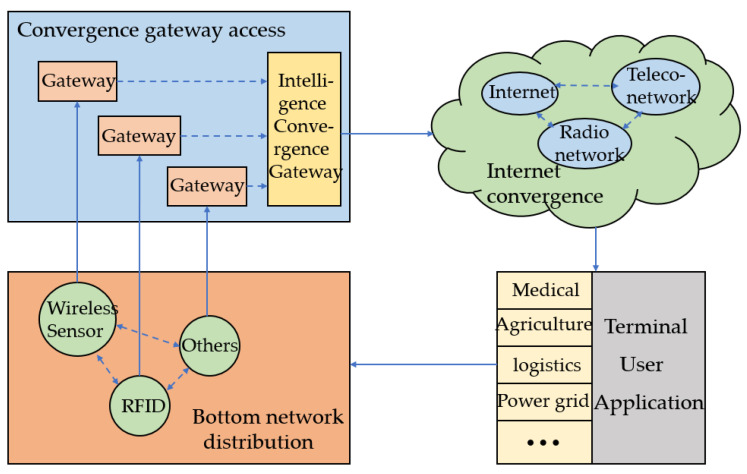
The Internet of Things system architecture of a wireless sensor network.

The IoT framework design of the remote sensor arrangement is shown in Figure 1, comprising four parts: the underlying network distribution, an intelligence convergence gateway, Internet convergence, and the terminal user application. As shown in Figure 1, a large number of IoT basic frameworks are specifically disseminated within a physical space, and the network distribution is shaped by comparing strategies relevant to its particular characteristics. The underlying network collects the exchanged data through RFID (Radio Frequency Identification), WSNs, wireless LAN, and other network technologies and transmits it to the intelligence convergence gateway. It accesses the network integration system through the intelligence convergence gateway.

**Figure 2:**
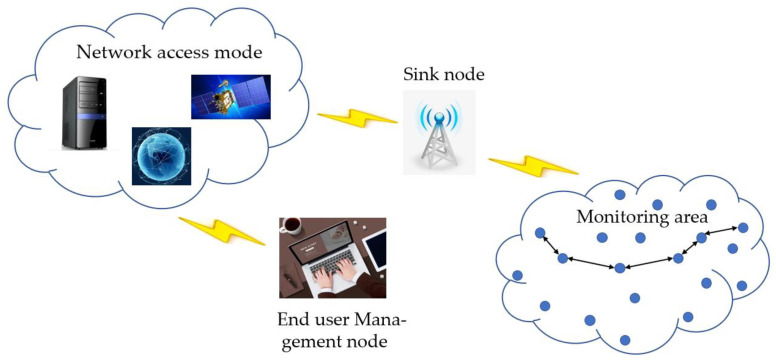
The architecture of a wireless sensor network based on the Internet of Things.

One of the important characteristics of the Internet of Things is to enable information exchange between sensors and networks. Each sensor is an object. Therefore, the IoT underlying network responsible for sensing and recording data must be able to reflect the characteristics of each object. The engineering of remote sensor arrangements based on the Internet of Things is shown in Figure 2. A large number of sensor hubs are connected within the checking region within the framework of an irregular spread or manual situation, and the organization is built through self-organization. The data in the area monitored by the sensor node finally arrive at the sink node through the multihop routing transmission of the nodes in the network. The data may be fused and compressed by multiple nodes during the transmission process, and they finally reach the management node of the terminal through a satellite, Internet, or wireless access server. Users can perform feedback operations such as configuration management, task publishing, and security control on WSNs through the management node.

### 3.2. The Positioning Mechanism of the Internet of Things Based on a Sensor Network

**Figure 3:**
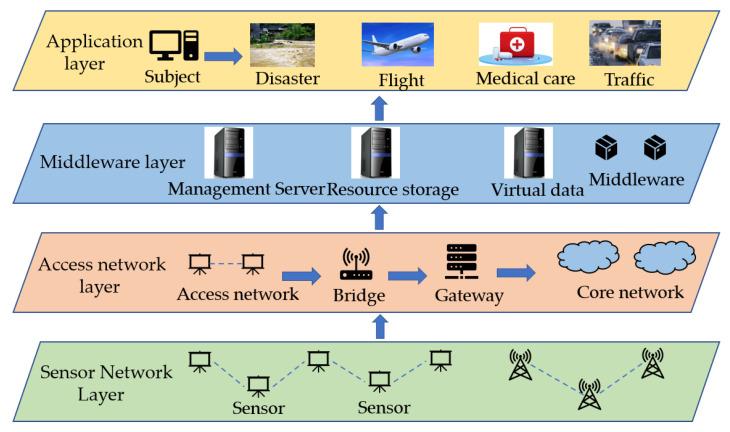
Positioning hierarchy of the Internet of Things based on sensor networks.

As shown in Figure 3, the positioning structure based on the Internet of Things has four levels. When wireless sensors are located in the Internet of Things system, canonical correlation analysis (CCA) can establish the mapping between two datasets and maximize the correlation between them, which is an effective modeling method. However, the CCA can only mine a linear relationship between the data, which is usually difficult to apply to the actual environment. The nonlinear kernel CCA (KCCA) is used to mine the nonlinear relationship between data to achieve the required nonlinear mapping, and the LE-KCCA sensor location algorithm has been proposed. However, the KCCA model adopts a unified global nonlinear mapping, without considering the local structural characteristics of the network [20]. On the other hand, the Internet of Things has a complex and topology-changeable form, which makes it extremely difficult to select the core parameters in the KCCA. The key to the effective construction of the KCCA model is the quality of the selection. The LPCCA method is recommended to mine the local topology data of signal space and physical space, and the LE-LPCCA sensor location model has been established on this basis. The node location accuracy can be greatly improved.

### 3.3. Establishment of the LE-LPCCA Positioning Algorithm Model Based on the Sensor Grid

In wireless sensor networks, the signal space data and physical space data collected by known sensors can be expressed as two sets of datasets [21], with X = [x1, x2,…, xn] ^p × N^ representing the signal strength received by n known nodes, where the dimension of each signal vector *x_i_* (I = 1, 2,…, n) is p, p is the number of Access Points (AP nodes), and Y = [y1, y2,…, yn] ^p × N^ represents the physical coordinate of the corresponding node. The direct neighbor q represents the direct neighbor of the sample data p, and the direct neighbor relationship is irreversible. If the direct neighbor of data point p is data point q, then the direct neighbor of data point q is not data point p, so q = 2 or 3. The primary task of building a positioning model is to establish the mapping between two datasets.

The CCA data mapping method based on a wireless sensor grid is a classical method used to construct the mapping between two groups of data. Its goal is to find two groups of basis vectors *w_x_* ∈ RP and *w_y_* ∈ Rq for X and Y, respectively, so that the correlation between the transformed data *w_x_*^T^ (*x_i_* − *x*) and *w_y_*^T^ (*y_i_* − *y*) can reach the maximum. Through the definition of correlation and several steps of mathematical derivation, the CCA can be expressed as the solution to the optimization problem:

(1) $$\underset{w_{x},w_{y}}{\max}w_{x}^{T}\overset{n}{\underset{i=1}{\sum }}\overset{n}{\underset{j=1}{\sum }}\left(x_{i}-x_{j}\right){\left(y_{i}-y_{j}\right)}^{T}w_{y}$$

(2) $$s\cdot t\cdot w_{x}^{T}\overset{n}{\underset{i=1}{\sum }}\overset{n}{\underset{j=1}{\sum }}\left(x_{i}-x_{j}\right){\left(x_{i}-x_{j}\right)}^{T}w_{x}=1$$

(3) $$w_{y}^{T}\overset{n}{\underset{i=1}{\sum }}\overset{n}{\underset{j=1}{\sum }}\left(y_{i}-y_{j}\right){\left(y_{i}-y_{j}\right)}^{T}w_{y}=1.$$

By solving the optimization problem (1), (2), and (3), we can obtain *w_x_* and *w_y_* and then transform the IoT data to the forms *w_x_*^T^ (*x_i_* − *x*) and *w_y_*^T^ (*y_i_* − *y*). After transformation, the correlation between the two datasets is the largest; however, if the mapping of the signal space to the physical space in the WSN is established based on the CCA, and the positioning model is built, only the linear correlation between two groups of data can be mined, and the local structure information of the network is not used. The wireless sensor algorithm model can measure the static strain of the structure, and the measurement data are better after the data fusion processing. Therefore, some scholars have used the algorithm model to collect the swing data of the large lifting pipe-laying vessel hook model and effectively verified the feasibility of the anti-swing control model.

In the signal space of wireless sensor networks, the ne (*i*) represents a set of node labels similar to the signal strength received by node *i*, that is, the subscript set of *x_i_*’s local nearest neighbor samples. In the physical space, the ne (*i*) represents the subscript set of nodes adjacent to the location of node *i*, where the local nearest neighbor is divided by the k-nearest neighbor method: if *x_j_* (*y_j_*) is the k-nearest neighbor sample of *x_i_* (*y_i_*), then *x_j_* (*y_j_*) is said to be the local nearest neighbor of *x_i_* (*y_i_*). The matrix elements are:

(4) $$S_{ij}{}^{X}=\exp\left(-∥x_{i}-x_{j}{∥}^{2}/t_{x}\right),j\in ne(i)/i\in ne(j)$$

(5) $$S_{ij}{}^{Y}=\exp\left(-∥y_{i}-y_{j}{∥}^{2}/t_{y}\right),j\in ne(i)/i\in ne(j).$$

It can be seen that in the positioning algorithm based on the wireless sensor networks, the larger the *S_ij_^X^*, the closer the distance between *x_i_* and *x_j_* (or *y_i_* and *y_j_*). If *x_i_* and *x_j_* (or *y_i_* and *y_j_*) are not in the neighborhood, the similarity is zero. *S_ij_^X^* depends on the layout of the sensor nodes; so, *S_ij_^X^* can change with the topology of the sensor network, showing a high degree of flexibility. The typical correlation in the local neighborhood of the sensor network can be defined as:

(6) $$w_{x}^{T}\cdot \overset{n}{\underset{j=1}{\sum }}S_{ij}^{X}\left(x_{i}-x_{j}\right)S_{ij}^{Y}{\left(y_{i}-y_{j}\right)}^{T}\cdot w_{y}$$

Therefore, the nonlinear problem of a wireless sensor global network can be decomposed into n local (quasi) linear subproblems. Conversely, the combination of these subproblems can be used as an approximation of the original problem. Therefore, after considering the local distribution characteristics of the data, the LPCCA can be described as the following optimization problem:

(7) $$\underset{w_{x},w_{y}}{\max}w_{x}^{T}\overset{n}{\underset{i=1}{\sum }}\overset{n}{\underset{j=1}{\sum }}S_{ij}^{X}\left(x_{i}-x_{j}\right)S_{ij}^{Y}{\left(y_{i}-y_{j}\right)}^{T}w_{y}$$

(8) $$s\cdot t\cdot w_{x}^{T}\overset{n}{\underset{i=1}{\sum }}\overset{n}{\underset{j=1}{\sum }}S_{ij}^{X^{2}}\left(x_{i}-x_{j}\right){\left(x_{i}-x_{j}\right)}^{T}w_{x}=1$$

(9) $$w_{y}^{T}\overset{n}{\underset{i=1}{\sum }}\overset{n}{\underset{j=1}{\sum }}S_{ij}^{Y2}\left(y_{i}-y_{j}\right){\left(y_{i}-y_{j}\right)}^{T}w_{y}=1.$$

### 3.4. The LE-RLPCCA Improved Positioning Algorithm Model Based on the Sensor Grid

In a complex or untrustworthy IoT environment, affected by network attacks, sensor hardware errors, environmental obstacles, and other factors, IoT data are prone to distortion or errors in the transmission or positioning process, which is different from the simple errors easily generated in ordinary networks [22]. The LE-RLPCCA localization algorithm optimizes the topology of the wireless sensor networks to a certain extent, so that the DKFCM-FSVM wireless sensor algorithm model proposed in this paper shows a high degree of data fusion and detection accuracy.

By characterizing the location algorithm LE-LPCCA of the wireless sensor networks, we can characterize the density based on the topology of the wireless sensor nodes:

(10) $$M_{i}^{X}=\frac{D_{i}^{X}}{\sum_{j=1}^{n}D_{j}^{X}};\;M_{i}^{Y}=\frac{D_{i}^{Y}}{\sum_{j=1}^{n}D_{j}^{Y}},$$

where $D_{i}^{X}$ describes the area density of node *i* in the signal space. The node density reflects the distribution of the wireless sensor nodes in the signal space and is usually expressed by the density matrix. If $D_{i}^{X}$ ($D_{i}^{Y}$) is larger, it means that the area density of node *i* is higher. Obviously, the larger the $M_{i}^{X}$ ($M_{i}^{Y}$), the greater the density of the wireless sensor network node *i* in the signal (physical) space. From this, we can obtain the density matrix of the signal space *M_x_* = diag [$M_{1}^{X}$, $M_{2}^{X}$,…, $M_{n}^{X}$] and the density matrix of the physical space *M_Y_* = diag [$M_{1}^{Y}$, $M_{2}^{Y}$,…, $M_{n}^{Y}$]. Substituting $M_{i}^{X}$ and $M_{i}^{Y}$ into Equation (10) to replace $S_{\mathrm{ij}}^{X}$ and $S_{\mathrm{ij}}^{Y}$, respectively, we can obtain the following optimization problems:

(11) $$\underset{w_{x},w_{y}}{\max}w_{x}^{T}\overset{n}{\underset{i=1}{\sum }}\overset{n}{\underset{j=1}{\sum }}M_{i}^{X}\left(x_{i}-x_{j}\right)M_{i}^{Y}{\left(y_{i}-y_{j}\right)}^{T}w_{y}$$

(12) $$s\cdot t\cdot w_{x}^{T}\overset{n}{\underset{i=1}{\sum }}\overset{n}{\underset{j=1}{\sum }}M_{i}^{X^{2}}\left(x_{i}-x_{j}\right){\left(x_{i}-x_{j}\right)}^{T}w_{x}=1$$

(13) $$w_{y}^{T}\overset{n}{\underset{i=1}{\sum }}\overset{n}{\underset{j=1}{\sum }}M_{i}^{Y2}\left(y_{i}-y_{j}\right){\left(y_{i}-y_{j}\right)}^{T}w_{y}=1.$$

Through further development and the combination of Equations (11)–(13), we have

(14) $$\begin{array}{l} \underset{w_{x},w_{y}}{\max}w_{x}^{T}XM_{XY}Y^{T}w_{y}\\ s\cdot t\cdot w_{x}^{T}XM_{XX}X^{T}w_{x}=1\\ w_{y}^{T}YM_{YY}Y^{T}w_{y}=1 \end{array}$$

By using the Lagrange multiplier method to solve the optimization problem (14), it is easy to obtain the following generalized eigenvalue equation of the RLPCCA:

(15) $$\left(\begin{array}{cc} 0 & XM_{X}Y^{T}\\ YM_{XX}X^{T} & 0 \end{array}\right)\left(\begin{array}{c} w_{x}\\ w_{y} \end{array}\right)=\lambda \left(\begin{array}{c} XM_{XX}X^{T}\\ YM_{YY}Y^{T} \end{array}\right)\left(\begin{array}{c} w_{x}\\ w_{y} \end{array}\right).$$

We solve (15) to obtain the basis vector group (*w_x_*, *w_y_*) and then transform the original data in the form of wxTx and wyTy,

(16) $$\mathrm{order}\;H={\left(XM_{XX}X^{T}\right)}^{-\frac{1}{2}}\left(XM_{X}X^{T}\right){\left(YM_{Y}Y^{T}\right)}^{-\frac{1}{2}}$$

(17) $$u={\left(XM_{XX}X^{T}\right)}^{\frac{1}{2}}w_{x},v={\left(YM_{Y}Y^{T}\right)}^{\frac{1}{2}}w_{y}\;.$$

Formula (15) can be summarized as:

(18) $$HH^{T}u=\lambda^{2}u$$

(19) $$H^{T}Hv=\lambda^{2}v\;.$$

## 4. The Application Model of Machine Learning in Wireless Sensor Networks

### 4.1. The Existing Problems of Machine Learning in Wireless Sensor Networks

Different from traditional network machine learning technology, a wireless sensor network algorithm has special technical requirements. In order to adapt to the limited environment, the design of traditional networks is based on the edge theory of “end-to-end”. The terminal system of wireless networks is closely related to the processing operations of all functions. The task of the intermediate nodes is to forward packets, which may not be a reasonable choice for wireless sensor networks [23]. Some protocols and algorithms designed for ad hoc networks may not meet the characteristics and application requirements of sensor networks. The role of node identification (such as the address) in sensor networks is not very important, because the applications do not care about the information on a single node, and the data processing, fusion, and calculation related to specific applications on intermediate nodes are also necessary. In dense wireless sensor networks, the distance between the adjacent nodes is very short [24]. The advantage of the low-power multihop communication mode is that the power consumption is reduced, and the concealment of the communication is improved. The interference of external noise in remote communication can be ignored. However, we should also be aware of the complexity of the communication protocol formulation caused by the multihop communication mode.

### 4.2. A Machine Learning Model of a Wireless Sensor Network

The machine-learning-based wireless sensor cascaded automatic encoder (SAE) can effectively extract low dimensional features of data, which is an important part of the feature extraction classification model SAEM.

The AE loss function includes the input and output mean square error constraints, weight attenuation constraints, and sparsity constraints:

(20) $$\min L(W,b)=\left[\frac{1}{m}\overset{m}{\underset{i=1}{\sum }}J\left(W,b;x^{\left(i\right)},y^{\left(i\right)}\right)\right]+\frac{\lambda }{2}\overset{n_{l}-1}{\underset{l=1}{\sum }}\overset{s_{l}}{\underset{i=1}{\sum }}\overset{s_{l+1}}{\underset{j=1}{\sum }}{\left(W_{ji}^{\left(k,l\right)}\right)}^{2}+\beta \overset{s_{2}}{\underset{j=1}{\sum }}KL(\rho //\rho j).\;$$

The wireless sensor automatic encoder trains the network parameters through the gradient descent algorithm to minimize the loss function. The main steps are as follows:

Step 1 For all *l*, let the matrix △*W*^(*k*,*l*)^ = 0 and the vector △*b*^(*k*,*l*)^ = 0.

Step 2 For *i* = 1 to *m*, calculate:

$$\begin{array}{cc} \Delta W^{\left(k,l\right)} & :=\Delta W^{\left(k,l\right)}+{𝛻}_{W^{\left(k,l\right)}}J\left(W,b;x^{\left(i\right)},y^{\left(i\right)}\right)\\ \Delta b^{\left(k,l\right)} & :=\Delta b^{\left(k,l\right)}+{𝛻}_{W^{\left(k,l\right)}}J\left(W,b;x^{\left(i\right)},y^{\left(i\right)}\right) \end{array}.$$

Step 3 Update the parameters:

$$\begin{array}{c} W^{\left(k,l\right)}=W^{\left(k,l\right)}-\alpha \left[\left(\frac{1}{m}\Delta W^{\left(k,l\right)}\right)+\lambda W^{\left(k,l\right)}\right],\\ b^{\left(k,l\right)}=b^{\left(k,l\right)}-\alpha \left[\frac{1}{m}\Delta b^{\left(k,l\right)}\right] \end{array}.$$

Step 4 Repeat Step 2 until it converges or reaches the maximum number of iterations, and output (*W*^(*k*,1)^, *b*^(*k*,1)^, *W*^(*k*,2)^, *b*^(*k*,2)^).

**Figure 4:**
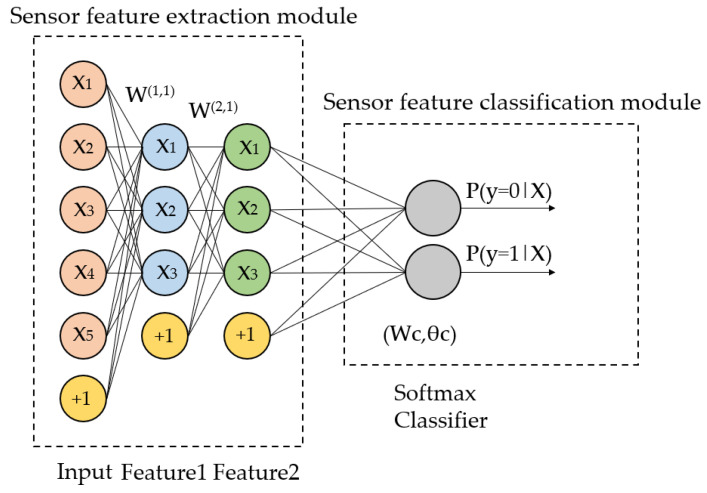
The feature extraction sensor classification model, SAEM, structure.

A few remote sensors arrange AEs to be cascaded to construct a multilayer neural arrangement [25]: the cascaded programmed encoder (SAE). The yield of the SAE can be seen as the included representation of the input data after different dimensionality decreases. The parameters of each layer of the SAE can be obtained through greedy training layer by layer (while training, the yield of the past hidden layer of the AE is utilized as the input for the following layer of the AE). The particular strategy was as follows:

We set the number of SAE hidden layers Nk of the sensor, utilized test x to train the primary AE to obtain its parameters, and the hidden layer yielded (1,2); we utilized (1,2) as the input, trained the next AE to obtain its parameters, and the hidden layer yielded (2,2), and so on. After the greedy training of the Nk AES layer by layer, we obtained the parameter to gather {(*W*^(*k*,*l*)^, *b*^(*k*,*l*)^) lk = 1,…, Nk}, and we took (*W*^(*k*,*l*)^, *b*^(*k*,*l*)^) as the association weight between the SAE layers, as shown in Figure 4.

### 4.3. The Data Fusion Algorithm of the Wireless Sensor Network Machine Learning Model

**Figure 5:**
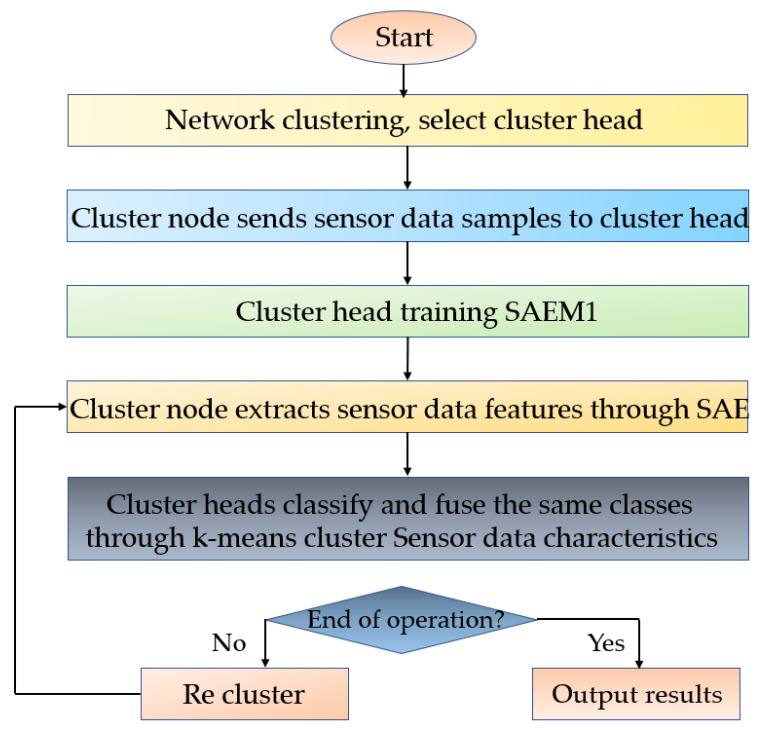
Wireless sensor machine learning SAEMDA1 algorithm flow.

After classification, the sensor hub data must be fused; so the model must be trained to compare the data extracted by the machine learning classification [26]. As the tests do not contain named data, an unsupervised data fusion calculation SAE-MDA1 based on SAEM1 and a supervised data fusion calculation SAE-MDA2 based on SAEM2 were designed. To facilitate the comparison and analysis, the WSN using the SAEMDA algorithm had the same network model as similar networks. First, we clustered the network through the clustering protocol and selected the cluster head node of each cluster; then, we ran the SAE-MDA1 algorithm (as shown in Figure 5) and the SAE-MDA2 algorithm. The main steps of the algorithm were as follows:

①SAEMDA1

**Step 1** Each cluster hub collected the sensor data and sent them to the comparison cluster head hub without processing.

**Step 2** The cluster head built and prepared the SAEM1 algorithm model based on the obtained cluster center data. At this time, the cluster head transmitted the SAE parameters to each cluster center, and the cluster head retained the K cluster parameters.

**Step 3** The cluster hub collected the detected data, employed the obtained SAE to extract the data features, and sent them to the cluster head.

**Step 4** The cluster head used the K-means cluster to classify features and fuse similar features according to Formula (21) and then sent them to the sink node

(21) $$f_{c}=\frac{1}{n_{c}}\overset{n_{c}}{\underset{i=1}{\sum }}a^{\left(N_{k},n_{l}\right)(i,c)},\;c=1,\cdots ,N_{c}.$$

②SAEMDA2

**Step 1** Each cluster head hub sent the cluster hub data table to the sink node.

**Step 2** The conglomeration hub developed and trained the SAEM2 with the training data containing named data as the input. At that point, the sink hub sent the SAE parameters back to each cluster hub and the SoftMax classifier parameters back to each cluster head node.

**Step 3** The cluster hub collected the detecting data and employed the SAE to extricate the features, sending them to the cluster head.

**Step 4** The cluster head employed the SoftMax classifier to classify the features and fused the similar features according to Equation (2); after that, it sent them to the sink node.

The SAEMDA2 had a lower time complexity because it had a shorter BP network and was trained in the convergence nodes, while the SAEMDA1 trained the SAEM1 network in the cluster head, and the cluster head needed to receive the original data sent by the cluster nodes as training samples, which increased the time and space complexity; in addition, the feature extraction and classification were performed in the cluster head in the SAEMDA1, while in the SAEMDA2, the cluster nodes performed the feature extraction locally, and the cluster head only performed the classification fusion, which reduced the amount of data transmission; therefore, the energy consumption of the SAEMDA2 was lower than that of the SAEMDA1.

For the SAEM1 and SAEM2 models of wireless sensor machine learning, the number of input layer units was the same as the collected data, and there were no specific parameters for organizing clusters and cluster centers. Therefore, the operation of the SAEMDA algorithm and the clustering convention of the wireless sensor machine-learning display were independent from each other.

## 5. An Engineering Mechanics Experimental Method Based on the Wireless Sensor Network

### 5.1. The Application of the Wireless Sensor Algorithm Model to an Engineering Mechanics Experiment

In 1996, U.S. scholar Strater Kiremidjian proposed the idea of replacing the wired system of an engineering mechanics experiment with wireless sensor technology, which introduced the application of wireless sensor network technology to the field of engineering mechanics experimentation and developed a real-time damage identification engineering structure health monitoring system. Based on the above work, Lynch and others developed a wireless sensor model using standard integrated circuits. The entire sensor node included an eight-bit microprocessor, and the detection unit was composed of a microacceleration chip. The integrated wireless sensor was verified in the laboratory. This laid a foundation for the application of engineering mechanics tests. Maser et al. designed the Wireless Global Bridge Evaluation and Monitoring System (WGBEMS) for monitoring the environment and performance of engineering mechanics in 1997. The sensor nodes in this system were composed of battery cells, microprocessors, small transponders, and sensors [27]. One area controller included in the system was placed on the bridge shore, and the other sensor nodes were placed on the bridge. The sensor node conducted data acquisition, filtering, identification, quantification, etc.

Some Chinese scientific research institutions and universities, such as Harbin University of Technology, Tsinghua University, the Ministry of Aerospace, and the Chinese Academy of Sciences, have initially researched sensors and wireless sensor networks [28]. As for the research and development of wireless sensors used for the mechanical monitoring of engineering structures, China is also in the initial stage. In general, the mechanical test monitoring of engineering structures belongs to a new research direction in the engineering field, and the application of wireless sensor networks to the mechanical monitoring of engineering structures is a hot spot of development. However, as wireless sensor network technology also belongs to a new discipline, it needs the cooperation of more disciplines to develop quickly, which may also slow the development of wireless sensor networks in the field of engineering mechanics experiments. Based on the application in the field of engineering structure monitoring, we tested the developed wireless sensor and its network system on representative structures such as reinforced concrete beams. This research focused on the testing and analysis of the wireless sensor algorithm model on the engineering mechanics test and further improved the wireless sensor hardware performance, software collaborative design, and other topics based on the analysis.

### 5.2. The Design of the Wireless Sensor Algorithm Model in an Engineering Mechanics Experiment

(1) The initial dataset partitioning algorithm based on wireless sensor density sensing

We assumed that the dataset of the designed mechanical test was *X* = {*x*_1_, *x*_2_,…, *x*_N_}, and each data point within the dataset was *x_i_* = {*x_i_*_1_, *x_i_*_2_,…, *x*_*i*M_}, where M represents the number of data test traits. The cluster center of the data is ordinarily determined by a neighbor test focused on the density, and the data centers with a high neighborhood density maintain a large distance between them. The local density of sample data *x_i_* is expressed as *ρ_i_*:

(22) $$\rho_{i}=\overset{N}{\underset{j=1}{\sum }}\exp\left[-{\left(\frac{∥x_{i}-x_{j}∥}{d_{c}}\right)}^{2}\right],$$

where ||*x_i_*−*x_j_*|| represents the Euclidean distance between *x_i_* and *x_j_*; *d_c_* is a predefined truncation distance, usually the first 1~2% of the truncation distance of all samples. The local density pi represents the number of data points whose distance from the sample data *x_i_* is less than *d_c_*. The more data points that are less than *d_c_* from the sample data *x_i_*, the greater the density value *ρ_i_*.

The characteristic distance represents the minimum distance between the engineering mechanics test sample data *x_i_* and the sample data with higher local density:

(23) $$\delta_{i}=\underset{x_{j}\cdot \rho_{j}>\rho_{i}}{\min}\left(∥x_{i}-x_{j}∥\right)\;,$$

where *δ_i_* represents the shortest distance between the engineering mechanics test sample data *x_i_* and the other high-density engineering mechanics test sample data. The sample data with high local density and large feature distance are more likely to become the cluster center.

The direct neighbor *q* represents the direct neighbor of the engineering mechanics test sample data *p*, which is defined as:

(24) $$q=\arg\underset{j:\rho_{p}>\rho_{i}}{\min}\left(∥x_{i}-x_{p}∥\right).$$

The engineering mechanics test sample data *x_i_* with the highest density has no direct neighbor, and the characteristic distance of such sample data is:

(25) $$\delta_{s}=\underset{s\neq i}{\max}\left(∥x_{i}-x_{s}∥\right).$$

**Figure 6:**
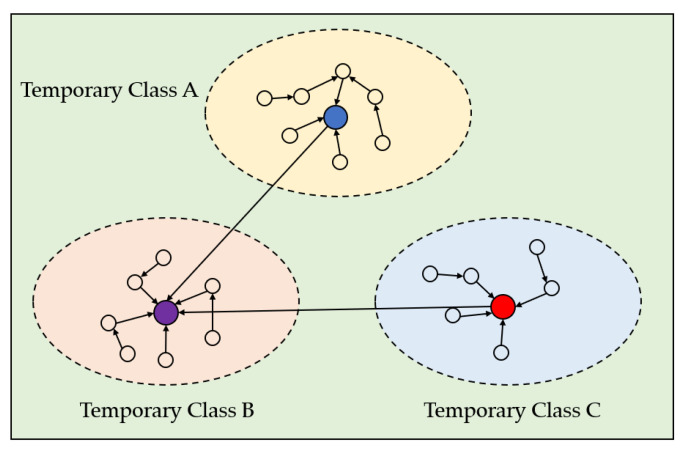
DAG-based temporary classes for the wireless sensor networks.

To build a directed acyclic graph (DAG) based on the wireless sensor networks, the test dataset of the construction machinery was divided into several levels according to the characteristic distance. If the dataset has a small feature distance, the data samples and their neighbors belong to the same category, while the feature points with a large feature distance and their neighbors do not belong to the same category. Therefore, after several iterations, the engineering mechanics test sensor dataset was divided into several temporary categories, as shown in Figure 6.

Finally, we clustered the number of remote sensor hubs C according to the fuzzy clustering calculation and intertwined the M temporary classes created to create the starting density-based parcel of the dataset. The clustering was conducted according to the following steps:

Step 1 Using the formula γi = ρiδi, we calculated the γ. The M transitory cluster centers were γ in descending order;

Step 2 After calculating the γ, we selected the cluster center of the primary C temporary classes as the introductory cluster center of the fuzzy clustering;

Step 3 We merged the tests within the remaining temporary classes into the primary C temporary classes where their neighbors were found, totaled the combination of the temporary classes, and framed the beginning parcel of the dataset.

(2) The fuzzy clustering algorithm based on sensor node density perception

The wireless strain sensor based on a fuzzy clustering algorithm was used to test the loading strain of reinforced concrete beams. During the test, in order to improve the accuracy of the strain data, the consistent measurement data with missing errors removed were fused to obtain more reliable measurement results than the arithmetic mean of the limited measurement data. We used the objective function definition of the DKFCM:

(26) $$J_{\mathrm{DKFCM}}(T,U,V)=\overset{c}{\underset{i=1}{\sum }}\overset{n}{\underset{j=1}{\sum }}u_{ij}^{m}t_{ij}^{p}∥\Phi \left(x_{j}-v_{i}^{\Phi }\right){∥}^{2}+\overset{c}{\underset{i=1}{\sum }}\eta_{i}\overset{n}{\underset{j=1}{\sum }}u_{ij}^{m}{\left(1-t_{ij}\right)}^{p}\;.$$

The objective function of the DKFCM algorithm was optimal. The absolute degree matrix *T* and the relative degree matrix *U* needed to meet the following requirements, respectively:

(27) $$t_{ij}={\left[1+{\left(\frac{∥\Phi \left(x_{j}\right)-v_{i}^{\Phi }{∥}^{2}}{\eta_{i}}\right)}^{\frac{1}{p-1}}\right]}^{-1}.$$

The local density of the wireless sensor nodes of the engineering mechanics test data and the initial division of the datasets were introduced into the fuzzy clustering algorithm, and the calculation formula for updating the cluster center was as follows:

(28) $$v_{i}^{\Phi }=\left(\overset{n}{\underset{j=1}{\sum }}u_{ij}^{m}t_{ij}^{p}\Phi \left(x_{j}\right)f_{i,j}\rho_{j}\right)\cdot {\left(\overset{n}{\underset{j=1}{\sum }}u_{ij}^{m}t_{ij}^{p}f_{i,j}\rho_{j}\right)}^{-1}$$

(29) $$D_{ij}^{2}=∥\Phi \left(x_{j}\right)-v_{i}^{\Phi }{∥}^{2}=K\left(x_{j},x_{j}\right)-2K\left(x_{j},{\tilde{v}}_{i}\right)+2K\left({\tilde{v}}_{i},{\tilde{v}}_{i}\right)$$

(30) $$K\left(x_{j},x_{j}\right)=\exp\left(\frac{∥x_{j}-x_{j}{∥}^{2}}{2\sigma^{2}}\right)=1$$

(31) $$\Phi \left(x_{j}\right)\cdot \left(\overset{n}{\underset{k=1}{\sum }}u_{ik}^{m}t_{ik}^{p}\Phi \left(x_{j}\right)f_{i,k}\rho_{k}\right)\cdot {\left(\overset{n}{\underset{k=1}{\sum }}u_{ij}^{m}t_{ij}^{p}f_{i,k}\rho_{k}\right)}^{-1}=\left(\overset{n}{\underset{k=1}{\sum }}u_{ik}^{m}t_{ik}^{p}K\left(x_{j},x_{k}\right)f_{i,j}\rho_{j}\right)\cdot {\left(\overset{n}{\underset{k=1}{\sum }}u_{ik}^{m}t_{ik}^{p}f_{i,j}\rho_{j}\right)}^{-1}$$

(32) $$K\left({\tilde{v}}_{i},{\tilde{v}}_{i}\right)={\left(\frac{\sum_{j=1}^{n}u_{ij}^{m}t_{ij}^{p}\Phi \left(x_{j}\right)f_{i,j}\rho_{j}}{\sum_{j=1}^{n}u_{ij}^{m}t_{ij}^{p}f_{i,j}\rho_{j}}\right)}^{2}=\frac{\left(\sum_{j=1}^{n}\sum_{k=1}^{n}u_{ij}^{m}t_{ij}^{p}u_{ik}^{m}t_{ik}^{p}K\left(x_{j},x_{k}\right)f_{i,j}\rho_{j}f_{i,k}\rho_{k}\right)}{{\left(\sum_{j=1}^{n}u_{ij}^{m}t_{ij}^{p}f_{i,j}\rho_{j}\right)}^{2}}\;.$$

### 5.3. Analysis of the Experimental Results of the Wireless Sensor Algorithm Model in the Engineering Mechanics Test

In the engineering mechanics intrusion detection experiment, the coverage of the wireless sensor sensing layer network was 100 m × 100 m, with 100 sensing nodes. We used the laptop as the gateway node, 10 detection nodes, and 90 ordinary nodes. The duration of each simulation was set to 200 s, and the average value of 20 simulation results was adopted for the experimental results. The WSN-DS dataset contained 386,563 records, and each sample of the dataset contained 20 attributes. We used 65% and 35% of the WSN-DS dataset as the training set and testing set, respectively.

We verified the remote sensor intrusion detection framework in the engineering mechanics in four ways: the true positive (TP) showed the extent of the abnormal behaviors accurately distinguished as abnormal behaviors; the false positive (FP) showed the extent of the normal behaviors wrongly distinguished as abnormal behaviors; the true negative (TN) showed the extent of normal behaviors accurately recognized as normal behaviors; the false negative (FN) demonstrated the extent of the abnormal behaviors wrongly recognized as normal behaviors. These provided the accuracy, the false positive rate, and the false negative rate, and by and large, the accuracy rate is the main assessment marker for remote sensor intrusion detection. To verify the remote sensor intrusion detection calculation, we chose the SVM, FCM-SVM, FCM-FSVM, and KFCM-FSVM as comparison algorithms. The DKFCM introduced data density and feature distance to improve the fuzzy clustering, which reduced the impact of the noise and the outliers on the clustering algorithm and accelerated the convergence speed of the clustering algorithm; the FSVM used the fuzzy membership obtained from the DKFCM as the fuzzy factor, which enhanced the objectivity of the fuzzy factor selection, improved the classification accuracy, and significantly improved the effectiveness of the intrusion detection in the WSN scenarios.

**Figure 7:**
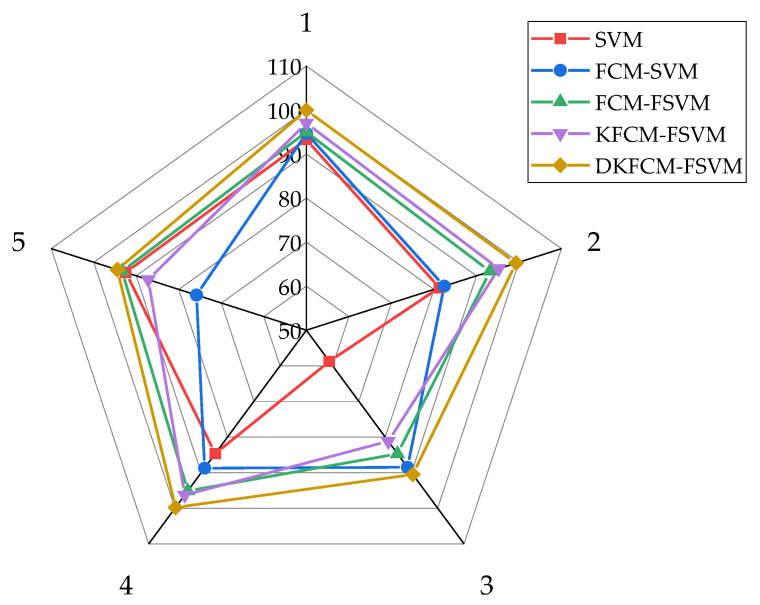
The accuracy of the wireless sensor network algorithm models.

The accuracy rate represents the proportion of the correct identification of the abnormal behavior and normal behavior. The comparison of the accuracy between the DKFCM-FSVM and the comparison calculations appears in Figure 7. Compared with the control calculations, the accuracy of the DKFCM-FSVM calculation proposed in this paper for recognizing five sorts of behaviors was 0.997, 0.992, 0.904, 0.996, and 0.946, individually, which achieved the highest accuracy in identifying the behaviors. The SVM calculation was faster and had the lowest accuracy, followed by the FCM-SVM calculation, which was prevalent in the SVM algorithm.

**Figure 8:**
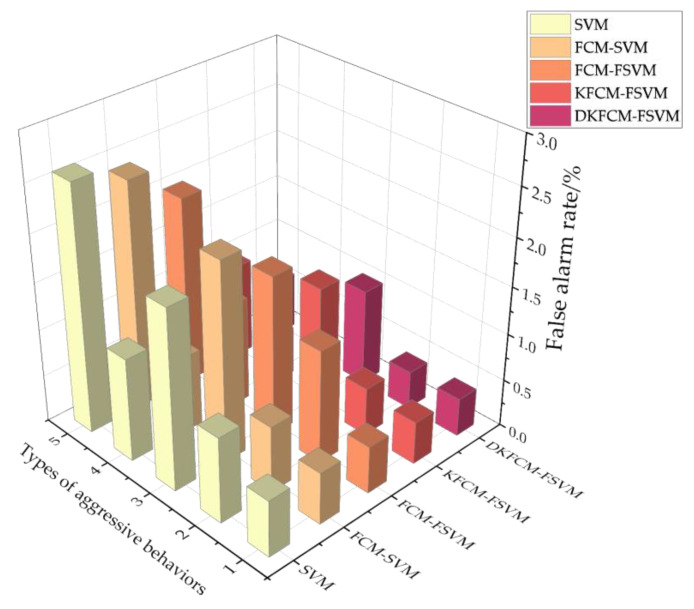
The false positive rate of the wireless sensor network algorithm models.

The comparison results of the false positive rate between the DKFCM-FSVM and the comparison calculations are shown in Figure 8. Compared with the control calculations, the DKFCM-FSVM’s false positive rates when identifying the five behaviors were 0.005, 0.004, 0.01, 0.003, and 0.006 respectively. It achieved the minimum false positive rate when identifying different behaviors. The false positive rate reflects the proportion of normal behaviors recognized as abnormal behaviors. This was because the FSVM used the fuzzy membership obtained from the DKFCM as the fuzzy factor, which enhanced the objectivity of the selection of the fuzzy factors and improved the classification accuracy, thus improving the effect of the intrusion detection in the WSN scenarios.

**Figure 9:**
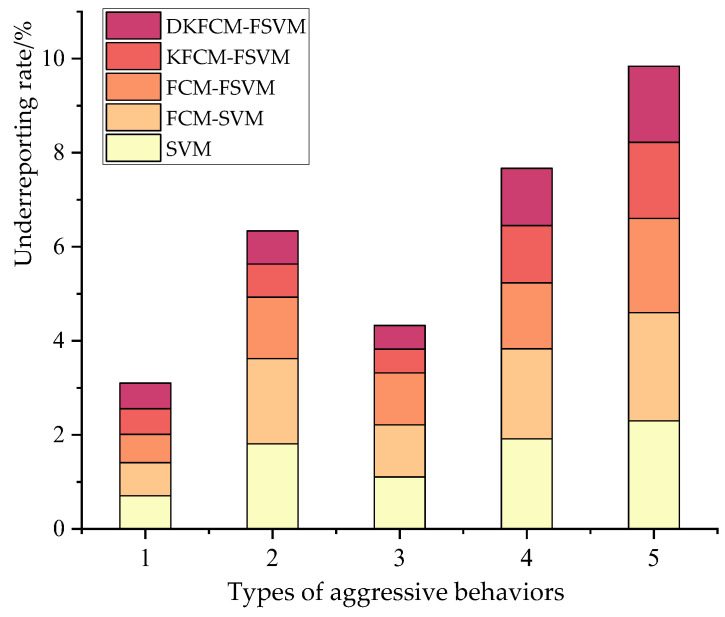
The false negative rate of the wireless sensor network algorithm models.

The false negative rate represents the proportion of abnormal behaviors that were not recognized and detected. The comparison results of the various algorithms are shown in Figure 9. Compared with the other calculations, the DKFCM-FSVM calculated and identified five kinds of behaviors, and the false negative rate of the intrusion detection was 0.004, 0.003, 0.003, 0.008, and 0.005 respectively, which was low as a whole. The SVM algorithm had a high false negative rate, followed by the FCM-SVM algorithm. In the WSN environment, most of the behaviors were normal, and the abnormal data generated by the intrusion attack accounted for a very small number. In most cases, they were linear and indivisible, and the kernel function needed to be mapped to the high-dimensional space. The detection effect of the SVM was poor. The FCM was sensitive to the selection of the initial clustering and unbalanced datasets, and the effect was not good. The DKFCM-FSVM algorithm effectively avoided the impact of the noise and the outliers on the classification results by introducing the data density and feature distance and achieved the optimal classification results.

**Figure 10:**
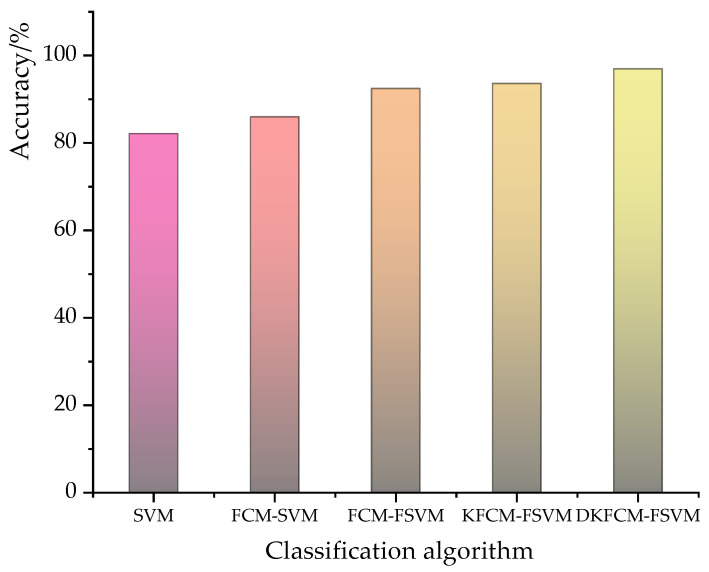
The overall detection accuracy of the wireless sensor network algorithm models.

The overall detection accuracy comparison between the DKFCM-FSVM and the control algorithms is shown in Figure 10. The overall detection accuracy of each algorithm was 0.8318, 0.8591, 0.9234, 0.9333 and 0.967 respectively. In the WSN-DS dataset, the accuracy of the DKFCM-FSVM intrusion detection was the best compared with the other algorithms, and its energy consumption was only slightly higher than that of the SVM. Therefore, the DKFCM-FSVM is applicable to the actual WSN scenario, thus improving the ability of WSNs to resist internal network attacks.

## 6. Conclusions

With the rapid development of sensor technology, embedded computing technology, the Internet of Things, and machine learning, wireless sensor networks have emerged. Due to their broad application prospects, wireless sensor networks have become a new research field in the 21st century. A large number of challenging problems have been raised for scientific and technological workers at the basic theory and engineering technology levels. The proposed wireless sensor algorithm model for engineering mechanics experiments involved wireless sensor networks, the Internet of Things, machine learning, and engineering mechanics experimental methods.

In this paper, the design and application of wireless sensor networks in engineering mechanics experimental methods were studied, and in-depth theoretical analysis and an experiment using the algorithm model was carried out. The following conclusions can be drawn from the current investigation.

This paper first analyzed the role of the Internet of Things in wireless sensor networks, studied the localization mechanism and the hierarchy of the Internet of Things based on wireless sensor networks, and improved the LE-RLPCCA localization algorithm model based on the sensor grids. Then the paper discussed the problems of machine learning in wireless sensor networks, constructed a sensor-based machine learning model, and designed a data fusion algorithm for a wireless sensor network’s machine learning model. This paper summarized the application of wireless sensors in engineering mechanics experiments, proposed an optimization algorithm model for the wireless sensors in engineering mechanics experiments, and analyzed the effectiveness of the algorithm model through engineering mechanics experiments. The test data showed that the average accuracy of the DKFCM-FSVM algorithm in detecting five behaviors was 0.997, 0.992, 0.904, 0.996, and 0.946, respectively, and the accuracy in detecting different behaviors was the best, which was 0.005, 0.01, 0.003, and 0.006 respectively. It achieved the lowest false positive rate in the detection of different behaviors, and the average false positive rate was 0.004, 0.003, 0.003, 0.008, and 0.005 respectively. Based on the research results, we found that the DKFCM-FSVM algorithm model of wireless sensor networks in engineering mechanics experiments was the optimal solution.

This research has a good reference value for the application of wireless sensor networks and the design of engineering mechanics experimental methods, and it is helpful for the further research of sensor technology.

## Figures and Tables

**Table 1:** Comparison of methods.

Author/Year	Purpose	Proposed Methodology	Key Parameters	Model
Luo et al. (2020)	Wireless sensor network intrusion detection	Fuzzy support vector machine	Accuracy and missing rate	DKFCM detection algorithm
Jiang et al. (2013)	Reduce the uneven distribution of sensor data	Sensor adaptive wireless routing strategy	Convergence time of Markov chains	An adaptive routing algorithm for wireless sensor networks
Liu et al. (2018)	Reduce the energy consumption of sensor networks	Wireless sensor network synchronization	Mean square error of clock offset	Clock synchronization algorithm model in wireless sensor networks
Zhang et al. (2020)	Reduce the energy consumption of multipath routing protocols	Routing algorithm with multiple quality of service constraints	Detection rate of sensors	Multi-stage sensor detection algorithm model
Guan et al. (2015)	Improve the efficiency of intrusion detection	The hybrid intrusion detection scheme	Running time and detection rate	ELM and SVM algorithm model
Mao et al. (2014)	Improve positioning accuracy	Wireless sensor network node location	Positioning error	SOAOLA location algorithm
Jiao et al. (2013)	Improve the positioning accuracy of the coal mine environment	Data representation of fuzzy expert knowledge	Kalman filter value	Takagi–Sugeno–Kang model
Proposed method	Improve positioning accuracy	Algorithm model of wireless sensor	Positioning accuracy	DKFCM-FSVM location algorithm

## Data Availability

The experimental data used to support the findings of this study are available from the corresponding author upon request.
